# Effect of Health Education on Healthy Nutrition and Physical Activity among Female Teachers Aged 40–60 Years in Asmara, Eritrea: A Quasiexperimental Study

**DOI:** 10.1155/2020/5721053

**Published:** 2020-09-25

**Authors:** Helen Gebretatyos, Soliana Amanuel, Lidia Ghirmai, Ghidey Gebreyohannes, Eyasu H. Tesfamariam

**Affiliations:** ^1^Department of Midwifery, School of Nursing, Asmara College of Health Sciences, Asmara, Eritrea; ^2^Asmara College of Health Sciences, Asmara, Eritrea; ^3^Biostatistics and Epidemiology Unit, Department of Statistics, College of Science, Mai Nefhi, Asmara, Eritrea

## Abstract

**Background:**

Middle age is a period in women's life where many changes occur in their bodies due to the decline of gonadotropins. As a result, they face various vasomotor, psychological, and somatic symptoms. Moreover, chronic illness such as diabetes, hypertension, heart diseases, and osteoporosis are prevalent at this time. Healthy nutrition and physical activity are effective factors to reduce the problems of menopause faced during middle age.

**Objective:**

This study aimed at assessing the effect of health education on healthy nutrition and physical activity among 40–60-year-old female teachers in elementary, junior, and secondary schools of Asmara.

**Method:**

A quasiexperimental design was used in this study. The data were collected from 99 middle-age female teachers who were selected by stratified random sampling. The intervention was conducted using lecture, brochure, and group discussion for a total of 3 hours. Data on physical activity and healthy nutrition were collecting using HPLP- II subscales physical activity and nutrition. Sociodemographic data were collected using a predesigned questionnaire. The effect of educational training at preintervention and postintervention was evaluated by the paired *t*-test and factorial mixed ANOVA using SPSS (version 22).

**Results:**

The mean score of practicing healthy diet and physical activity at preintervention and postintervention was 27/44 (SD = 4.20) and 31/44 (SD = 5.36), respectively. A significant difference in the scores of practicing healthy diet and physical activity was observed after the educational intervention (MD = 4.06, 95% CI 2.95–5.17, *p* < 0.0001). The effectiveness of health education was seen across the categories of age, educational level, and occupational level where none of them showed significant interaction, displaying similar effect of educational intervention across all categories of the demographic variables.

**Conclusion:**

The structured educational intervention was beneficial for the studied women in changing their practice on healthy nutrition and physical activity.

## 1. Introduction

Menopause is an important milestone in women's life, and usually, it occurs naturally at the age of 51 on average as it has great variation depending on the age of menarche, ethnicity, history of prior contraceptive use, current smoking status, and genetic factors, yet its effects are physical and also psychological [1, 2]. The feminine hormones (estrogen and progesterone) have the capacity to shield women from various diseases, where the male counterpart could not be shielded [3]. Gradual decline and cessation of secretion of these hormones occur at some point in women's life, resulting in various effects on their health and well-being. Some of the health problems seen during the menopausal period are osteoarthritis, osteoporosis, skin changes, cardiovascular accidents, and stroke [4]. The most cost-effective way to avoid, as well as alleviate, many of the health problems is by improving one's lifestyle.

Healthy lifestyle is an effective intervention to decrease menopausal complications and gives a sense of self-sufficiency to women in perimenopause and postmenopause. Such a healthy lifestyle would also have an added economic benefit through reduced expenditure on palliative treatments. According to Pender, healthy lifestyle behaviors include self-actualization, health responsibility, exercise, nutrition, interpersonal relations, and stress management [5]. Physical activity and nutrition are the most important part of this healthy lifestyle behavior in menopausal women as they directly influence menopausal symptoms and associated health problems [6–8]. The integration of physical activity and healthy nutrition in women's life would be prominent if the health awareness of women initiated during perimenopause and early postmenopause [9].

On a wider scale, physical activity and healthy nutrition are essential behaviors for middle-age women as they can prevent or reduce certain conditions that may develop during and after menopause, including, but not limited to, obesity, type II diabetes, heart disease, certain types of cancer, and osteoporosis [10–19]. Besides, physical activity and healthy nutrition play a key role in making the transition through menopause easier through minimizing climacteric symptoms [6, 8, 9, 20, 21]. These symptoms affect some women more than others. Therefore, they face a devastating effect in their social life, personal life, and quality of life [22]. Furthermore, being a teacher adds its toll as teaching demands patience, high concentration, stable mood, and good memory [5].

Healthy nutrition indicates being aware of the calorie content of food and beverages per portion consumed and remembering to consume less energy giving food if you are less active. Various food guides recommend the consumption of a diet rich in vegetables, fruits, whole-grains, fiber, and fish [23, 24]. Furthermore, eating saturated fat, sugar, and salt should be limited; in turn, intake of dairy products should be increased as its effect on decreasing osteoporosis is high during the postmenopausal time. Physical activity is any bodily movement produced by skeletal muscle that requires energy expenditure [25]. Menopausal guides recommend involving in moderate activity for at least 30 min for five days or more per week [26–29]. This activity includes aerobics, stretching, and weight bearing.

Despite this fact among women in the middle age (40–60 years), 30% report no physical activity at all, and the prevalence of inactivity progressively increases with age [10, 30]. Likewise, fitness levels decline 1% to 2% per year during the postmenopausal years [15]. Moreover, women lack the necessary awareness on the dietary changes required during the menopausal time [31, 32]. Any attempt of lifestyle modification must include behavioral changes to implement the new diet and physical activity regimen. Health education strategy is one of the alternative strategies for improving women's behavior on healthy eating and being active [33]. Hence, the main objective of this study is to equip middle-age teachers with the necessary knowledge to change their behavior on physical activity and healthy nutrition. According to the results of this study, replication of this study will be useful in improving the status of Eritrean women's quality of life at menopause and beyond.

## 2. Materials and Methods

### 2.1. Design, Study Setting, and Population

A semiexperimental design with preintervention and postintervention (3 months later) was employed in this study. This was conducted from June to October 2018 in Asmara, Eritrea. Asmara is the capital city and largest settlement in Eritrea, home to a population of 416,367 people according to records in the municipality of Asmara. There are about 75 schools in Asmara; out of this, there are 37 elementary, 28 junior, and 11 secondary schools. At the time of the study, there were 677 female teachers aged 40 to 60 years working in elementary (*n* = 562), junior (*n* = 87), and secondary (*n* = 28) schools.

### 2.2. Sampling

Sample size was calculated using a G-power (Version 3) calculator based on priori by giving *α*, power, and effect size. Matched pairs were selected as the test family in the test software. The input required were tails (two), effect size (0.3), actual power (0.80), and level of significance (0.05) which resulted in a sample size of 90 women. Considering 10% dropout from follow-up, finally, 99 females were found to be adequate. Stratified random sampling was used to select female teachers. First, a stratum was formulated using the occupational level, and then, simple random sampling was used to select female teachers proportionally. The selected size of sample of female teachers from elementary, middle, and secondary levels was 82, 13, and 4, respectively.

### 2.3. Intervention

  Step 1: Permission was attained from the Research Ethical Committee of Asmara College of Health Sciences and zonal office of the Ministry of Education and each school authority. Then, those teachers who were aged from 40 to 60, without sight difficulty and who were present in their working place during the data collection time were approached. Among the females, only those who gave informed written consent participated in the study. Participants were first given a preinterventional test on July 01, 2018. Then, the 99 participants were grouped into 10 groups in which nine groups contained 10 teachers except one.
  Step 2: At this time, each group was called to the premises of Asmara College of Health Sciences for provision of the training. This training was given for 3 days for a total of 3 hours for each group, and the total time taken for the training was one month. The training was given by an expert midwife based on teaching manual prepared by the investigator from several sources [26–29]. The training mainly focused on the definition of menopause, age at menopause, symptoms of menopause, physical activity and its benefit in reducing symptoms of menopause, nutrition, and what improvements should be employed when women reach menopause and postmenopause. It was given in the form of lectures, and group discussion was conducted based on the teaching manual. After the training, all study participants were sent with handouts and brochure which have the same content as the teaching manual [26–29].
  Step 3: After 3 months, all study participants were called for a postintervention test, and 98 came which made the response rate 98.9%.

### 2.4. Instrument

Walker and others developed the health promotion lifestyle profile (HPLP) in 1987 in English that evaluates health promotion lifestyle-related behaviors. The original HPLP includes 48 items on six subscales: self-actualization, health responsibility, exercise, nutrition, interpersonal support, and stress management. The HPLP demonstrated strong validity and reliability [34]. The alpha reliability coefficient for the total scale is 0.922; the alpha reliability coefficient subscale ranged from 0.702 to 0.904 [34]. In 1990, the HPLP was translated from English to Spanish and tested with 485 Hispanic adults, showing that the Spanish version had sufficient validity and reliability [35]. The alpha reliability coefficient for the total scale is 0.93; alpha reliability coefficient subscale range from 0.70 to 0.87 [35]. In 1995, the original 48-item HPLP was revised and resulted in the 52-item HPLP-II. The revised 52-item instrument also includes six dimensions: health responsibility, physical activity, nutrition, spiritual growth, interpersonal relations, and stress management. Three of the six original dimension names were changed. The subscale self-actualization was renamed spiritual growth; interpersonal support was renamed interpersonal relations; and exercise was renamed physical activity [36]. In this study, HPLP-II, subscales physical activity, and nutrition were used for data collection at pre- and postintervention together with a structured questionnaire for the sociodemographic variables. This was performed after back-to-back translation to Tigrigna. Then, content and face validity were assured through evaluation of the questionnaire by various experts who are 01 with PhD in obstetrics and gynecology in nursing, 02 with MSc in midwifery, and 01 professor in geophysics, 01 statistician with MSc in biostatics and epidemiology. The internal consistency was calculated using Cronbach alpha (0.754).

### 2.5. Statistical Analysis

The collected data were coded, double-entered, cleaned, and analyzed using Statistical Package for Social Sciences (SPSS, version 22.0). The Kolmogorov–Smirnov test and Fisher's measures of skewness and kurtosis were used to check the normality of the data. To describe and summarize the study results, mean (SD) for continuous variables and frequency (percentages) for categorical variables were used. The mean score of practice on physical activity and healthy nutrition was computed on preintervention and postintervention. The change of scores at the two time points was investigated using the *t*-test. Factorial mixed ANOVA was used to determine effectiveness of health education on physical activity and healthy nutrition across the categories of demographic variables. The results were presented using tables. A statistical significance was considered at a *p* value of less than or equal to 0.05 except for Box M *p* value of less than or equal to 0.0001.

## 3. Results

Hundred and ten middle-age teachers were approached in the study, out of which 11 excluded due to failure to give consent and unwillingness to participate in this study. Finally, 99 middle-age women participated in which one lost follow-up that made the response rate 98.9%.

### 3.1. Sociodemographic Characteristics

The sociodemographic variables are shown in Table 1. The mean age of the study participants was 48.97 (SD = 5.47) years, and 77.8% of the respondents were married. Regarding educational status of the respondents, 80 (80.8%) of the respondents had certificate and the remaining had diploma and degree. Most (82.8%) of the study participants were working in elementary schools. The majority (68.7%) of the study participants had a monthly income of $133.3–$166.6.

### 3.2. Respondents' Reported Practice Level before and after Intervention

In Table 2, the proportion of women who reported regularly/often is presented. The percent of women who follow planned exercise was 55.6% at preintervention and increased to 69.4% at postintervention. Women that included exercise in their usual activity were 71.7% at preintervention and became 79.6% at postintervention. Participants consumption of healthy diet had increased in most of the parameters except in the consumption of carbohydrate which remained the same (*n* = 44) before and after intervention.

### 3.3. Effect of Health Education on Practice

A paired *t*-test was used to assess the effect of educational intervention on the practice of healthy nutrition and physical activity through time (Table 3). The mean score at postintervention (*M* = 31/44, SD = 5.36) was significantly greater (*p* < 0.0001) than that of preintervention (*M* = 27/44, SD = 4.2). The mean difference of scores was 4.06 (95% CI: 2.95, 5.17).

### 3.4. Effectiveness of Educational Intervention on Practice across Background Characteristics

Factorial mixed analysis of variance (ANOVA) was conducted to assess the impact of educational intervention on the scores of practice on healthy diet and physical activity across two time points (preintervention and postintervention) by age, educational level, and occupational level (Table 4). There was no significant interaction between age and time (preintervention and postintervention) (Wilk's Lambda = 0.992, *p*=0.391), educational level and time (Wilk's Lambda = 0.999, *p*=0.742), and between occupational level and time (Wilk's Lambda = 0.998, *p*=0.665).

## 4. Discussion

Respondents reported practice on healthy nutrition and physical activity before and after intervention.

It is not clear if menopausal transition alone brings weight gain in women though physiological withdrawal of estrogen has proven to bring about changes in fat distribution. This results in greater fat deposition in the upper body than lower body in postmenopausal women [37]. Even though fitness has a genetic contribution, physical activity habits are the primary determinants of fitness in adults, and changes in physical activity result in changes in fitness and subsequent mortality [10]. In this study, female teachers that follow planned exercise were slightly below half at preintervention; however, at postintervention more than half of the study participants started to follow planned exercise. Likewise, study participants that included exercise in their daily activity were 71.7% before intervention and became 79.6% at after intervention. This finding is important because women that incorporate physical activity in their daily activity are more likely to engage and follow it sustainably without interruption. Hence, it reduces the morbidity and mortality related to lack of fitness. Moreover, women who exercise might have lower episodes of vasomotor symptoms during menopause than those who do not exercise [8, 9, 20]. Especially for middle-age teachers, symptoms of menopause affect their performance as it adds to the stress of teaching, so adapting exercise might help them in tackling this problem. There was a similar finding in an Iranian study in which most of the study participants changed their behavior regarding exercise [38]. This study in Iran also reported that women's level of balanced exercise increased after intervention in order to reduce their risk of osteoporosis and fall accidents.

Besides, in this study, 67% of the participants started to exercise vigorously for 20 minutes or more at least three times per week after the provided training that was 18% before intervention. This is an indication that the educational intervention was helpful on enlightening women on what type of exercise and to how much they should perform it. Furthermore, almost all of the study participants were engaged in light to moderate physical activity after the educational intervention.

In this study, participants' consumption of healthy diet increased at postintervention, even though intake of carbohydrate rich food remained the same (*n* = 44) before and after intervention. This result may be due to the availability and reasonable prices of carbohydrates, so people tend to consume them. Women's intake of two to three servings of milk and milk products each day increased from 30.3% at preintervention to slightly less than half of the study participants at postintervention. Increased calcium intake coupled with essential amount of vitamin D is critical for the prevention of osteoporosis in middle-age women [39]. In the United States, osteoporosis affects over 20 million Americans [40]. This affects the quality of life that negatively influences the morbidity and mortality of women at postmenopause.

In this study, women tend to choose a diet low in saturated fat at postintervention (44.4% vs. 54.1%). Decreasing saturated fat intake might decrease the risk of cardiovascular disease (CVD) as CVD is a very common problem in middle-age women and more than half of sudden deaths are due to CVD [41]. Moreover, increasing consumption of fruits and vegetables can diminish this risk [17]. According to the intervention provided in this study, the study participants' consumption of 2–4 servings of fruit and 3-5 servings of vegetable per day increased at postintervention when compared to preintervention. The Canadian food guide recommends for the consumption of seven servings of vegetable and fruit per day for women in menopause and postmenopause [23]. Similarly, in a study conducted in Egypt, the study participants' food habit significantly changed after intervention with increased consumption of calcium, protein, fish, legumes, vegetables, and fruits but there was no change in carbohydrate intake [39].

In this study, the mean score of practicing healthy diet and physical activity significantly increased after intervention as compared to that of preintervention. This is promising for middle-age Eritrean women, promoting healthy diet and physical activity could be easily adapted as their behavior, hence decrease the risk of menopausal complications and improve their quality of life. Studies conducted in Egypt and India regarding the effect of health education on health promoting behavior, which incorporates healthy eating and physical exercise, showed significant increase in the health-promoting behavior of the interventional group after the health education [42, 43]. Similarly, another Egyptian study has shown significant increase in the physical activity of the study participants after educational intervention [44].

The educational intervention had the same effect on healthy diet and physical activity across the categories of age, educational level, and occupational level in this study, whereas in a study conducted in Egypt, there was a significant difference in the effect of educational intervention across occupational categories [39]. The current study could not make comparisons across various categories of occupation, instead with the levels of occupation for the study was conducted in an educational setting only rather than in a community.

## 5. Study Limitation

The main limitation of this study was lack of the control group and usage of a questionnaire for assessment of behavioral change in physical activity and nutrition, but generally, women in Eritrea barely receive information regarding healthy eating and physical activity during middle age. This finding can help to generalize for the population of middle-age women in Eritrea.

## 6. Conclusions

This study aimed at investigating the effect of educational intervention on healthy nutrition and physical activity among middle-age female teachers. The study results demonstrated a positive impact of the educational intervention on healthy nutrition and physical activity. It is a fact that women living longer than men is only benefitting when they are aware about the type of food and calories suitable for them according to their age. Furthermore, they need to be knowledgeable about the recommended daily physical activity and its benefits.

## Figures and Tables

**Table 1:** Sociodemographic characteristics of the study participants.

Variable	Number	Percent
Age (mean = 48.97, SD = 5.47)		

*Educational level*		
Certificate	80	80.8
Diploma	16	16.2
Degree	3	3.0

*Occupation*		
Elementary school teacher	82	82.8
Junior school teacher	13	13.2
High school teacher	4	4.0

*Marital status*		
Married	77	77.8
Single	11	11.1
Divorced	8	8.1
Separated	3	3.0

*Ethnicity*		
Tigrigna	98	99.0
Shao	1	1.0

*Religion*		
Orthodox	81	81.8
Muslim	4	4.0
Catholic	8	8.2
Protestant	3	3.0
Other	3	3.0

*Gross monthly salary*		
<66.67$	3	3.0
66.67$–133.27$	13	13.1
133.3$–166.6$	68	68.7
>166.67$	15	15.2

**Table 2:** Percentage distribution of women who regularly/often practice healthy nutrition and physical activity at preintervention and postintervention.

Practice	Preintervention (*n* = 99)	Three-month follow-up (*n* = 98)
	Regularly/often *n* (%)	Regularly/often *n* (%)
Choose a diet low in saturated fat	44 (44.4)	53 (54.1)
Follow a planned exercise program	55 (55.6)	68 (69.4)
Limit use of sugar and food containing sugar	44 (44.4)	49 (50.0)
Exercise vigorously for 20 or more minutes at least 3 times per week	18 (18.2)	61 (61.6)
Eat 6–11 servings of bread, cereal, and pasta each day	41 (41.4)	41 (41.8)
Take part in light to moderate physical exercise	77 (77.8)	84 (84.8)
Eat 2–4 serving of fruit each day	25 (25.3)	56 (57.1)
Eat 3–5 serving of vegetable each day	53 (53.5)	65 (66.3)
Eat 2‐3 serving of milk or milk products each day	30 (30.3)	41 (41.8)
Exercising during usual daily activities	71 (71.7)	78 (79.6)
Eat 2‐3 serving from meat, poultry, fish, dried beans, egg and nuts each day	38 (38.4)	43 (43.9)

**Table 3:** Paired *t*-test at preintervention and postintervention.

Variable	M (SD)	MD (95% CI)	*p* value
Preintervention	27/44 (4.20)	4.06 (2.95, 5.17)	<0.0001
Three-month follow-up	31/44 (5.36)		

**Table 4:** Effectiveness of educational intervention on practice across background characteristics.

Characteristics	Preintervention M (SD)	3-month follow-up M (SD)	Box M *p* value	Wilk's Lambda	*p* value
*Age*					
40 to 50	27.03 (4.29)	31.50 (5.34)	0.984	0.992	0.391
51 to 60	27.74 (4.09)	31.20 (5.41)			

*Occupational level*					
Elementary	26.56 (3.52)	30.73 (5.28)	0.055	0.998	0.665
Junior or secondary	30.59 (5.57)	34.12 (4.96)			

*Educational level*					
Certificate	26.41 (3.61)	30.56 (5.24)	0.263	0.999	0.742
Diploma or degree	30.79 (4.76)	34.47 (4.77)			

## Data Availability

The complete dataset used and/or analyzed during the current study is available from the corresponding author and can be accessed upon reasonable request.

## References

[1] Gold E. B. (2001). Factors associated with age at natural menopause in a multiethnic sample of midlife women. *American Journal of Epidemiology*.

[2] Wang M., Gong W.-W., Hu R.-Y. (2018). Age at natural menopause and associated factors in adult women: Findings from the China Kadoorie biobank study in Zhejiang rural area. *PloS One*.

[3] Freeman E. W., Sammel M. D., Lin H., Gracia C. R. (2012). Anti-mullerian hormone as a predictor of time to menopause in late reproductive age women. *The Journal of Clinical Endocrinology & Metabolism*.

[4] Harlow S. D., Gass M., Hall J. E. (2012). Executive summary of the Stages of reproductive aging workshop + 10. *Menopause*.

[5] Sorour A. S., Wafik Kamel W., Abd El-Aziz E. M., Aboelseoud V. (2014). Health promoting lifestyle behaviors and related risk factors among female employees in Zagazig city. *Journal of Nursing Education and Practice*.

[6] Dormire S., Howharn C. (2007). The effect of dietary intake on hot flashes in menopausal women. *Journal of Obstetric, Gynecologic & Neonatal Nursing*.

[7] Skrzypulec V., Dąbrowska J., Drosdzol A. (2010). The influence of physical activity level on climacteric symptoms in menopausal women. *Climacteric*.

[8] Ueda M. (2004). A 12-week structured education and exercise program improved climacteric symptoms in middle-aged women. *Journal of Physiological Anthropology and Applied Human Science*.

[9] Kim M.-J., Ahn Y., Yim G., Park H.-Y. (2014). Association between physical activity and menopausal symptoms in perimenopausal women. *BMC Women’s Health*.

[10] Blair S. N. (1995). Changes in physical fitness and all-cause mortality: A prospective study of healthy and unhealthy men. *JAMA: The Journal of the American Medical Association*.

[11] Blümel J. E., Fica J., Chedraui P. (2016). Sedentary lifestyle in middle-aged women is associated with severe menopausal symptoms and obesity. *Menopause*.

[12] Church T. S., Earnest C. P., Skinner J. S., Blair S. N. (2007). Effects of different doses of physical activity on cardiorespiratory fitness among sedentary, overweight or obese postmenopausal women with elevated blood pressure. *JAMA*.

[13] Danaei G., Vander Hoorn S., Lopez A. D., Murray C. J. L., Ezzati M. (2005). Causes of cancer in the world: comparative risk assessment of nine behavioural and environmental risk factors. *The Lancet*.

[14] Dubnov-Raz G., Pines A., Berry E. M. (2007). Diet and lifestyle in managing postmenopausal obesity. *Climacteric*.

[15] Fleg J. L., Morrell C. H., Bos A. G. (2005). Accelerated longitudinal decline of aerobic capacity in healthy older adults. *Circulation*.

[16] Kruger J., Ham S. A., Kohl H. W. (2005). Trends in leisure-time physical inactivity by age, sex, and race/ethnicity-United States, 1994–2004. *Morbidity and Mortality Weekly Report*.

[17] Liu S., Manson J. E., Lee I.-M. (2000). Fruit and vegetable intake and risk of cardiovascular disease: the Women’s Health Study. *The American Journal of Clinical Nutrition*.

[18] Saris W. H. M., Blair S. N., van Baak M. A. (2003). How much physical activity is enough to prevent unhealthy weight gain? Outcome of the IASO 1st Stock Conference and consensus statement. *Obesity Reviews*.

[19] Stojanovska L., Apostolopoulos V., Polman R., Borkoles E. (2014). To exercise, or, not to exercise, during menopause and beyond. *Maturitas*.

[20] Santoro N., Epperson C. N., Mathews S. B. (2015). Menopausal symptoms and their management. *Endocrinology and Metabolism Clinics of North America*.

[21] Waszak M., Cieślik K., Grabowska M. (2007). Physical activity as a modifier of the course of menopause. *Studies in Physical Culture & Tourism*.

[22] Williams R. E., Levine K. B., Kalilani L., Lewis J., Clark R. V. (2009). Menopause-specific questionnaire assessment in US population-based study shows negative impact on health-related quality of life. *Maturitas*.

[23] Jessri M., L’Abbe M. R. (2015). The time for an updated Canadian food Guide has arrived. *Applied Physiology, Nutrition, and Metabolism*.

[24] Reid R., Abramson B. L., Blake J. (2014). Managing menopause abstract and summary statement. *Journal of Obstetrics and Gynaecology Canada*.

[25] Thompson P. D., Buchner D., Pin˜a I. L. (2003). Exercise and physical activity in the prevention and treatment of atherosclerotic cardiovascular disease. *Circulation*.

[26] Lark S. M. (1990). *Dr. Susan Lark’s the Menopause Self Help Book: A Woman’s Guide to Feeling Wonderful for the Second Half of Her Life*.

[27] Neves-e-Castro M., Birkhauser M., Samsioe G. (2015). EMAS position statement: The ten point guide to the integral management of menopausal health. *Maturitas*.

[28] Seaman B., Eldridge L. (2008). *The No-Nonsense Guide to Menopause: A Comprehensive Resource with Simple, Unbiased Advise on Managing This Important Life Stage*.

[29] Wingert P., Kantrowitz B. (2006). Is it hot in here? Or is it me?. *The Complete Guide to Menopause*.

[30] Bouchard C., Perusse L. (1994). Heredity, activity level, fitness, and health. *Physical Activity, Fitness, and Health: International Proceedings and Consensus Statement*.

[31] Hasan P. A. B., Abbasi Z. (2006). Education on middle-aged women’s knowledge and attitude towards menopause in Mashhad.

[32] Sultan S., Sharma A., Jain N. K. (2017). Knowledge, attitude and practices about menopause and menopausal symptoms among midlife school teachers. *International Journal of Reproduction, Contraception, Obstetrics and Gynecology*.

[33] Yazdkhasti M., Simbar M., Abdi F. (2015). Empowerment and coping strategies in menopause women: A review. *Iranian Red Crescent Medical Journal*.

[34] Walker S. N., Sechrist K. R., Pender N. J. (1987). The health-promoting lifestyle profile: Development and psychometric characteristics. *Nursing Research*.

[35] Walker S. N. (1990). A Spanish language version of the health-promoting lifestyle profile. *Nursing Research*.

[36] Frank-Stromborg M., Olsen S. (2004). *Instruments for Clinical Health-Care Research*.

[37] Genazzani A. R., Gambacciani M. (2006). Effect of climacteric transition and hormone replacement therapy on body weight and body fat distribution. *Gynecological Endocrinology*.

[38] Taherpour M., Sefidi F., Afsharinia S., Hamissi J. H. (2015). Menopause knowledge and attitude among Iranian women. *Journal of Medicine and Life*.

[39] Gamal A. M., Rashed A. B. (2015). Effect of systematic health education on perimenopausal rural women’s knowledge and practices regarding osteoporosis. *IOSR Journal of Nursing and Health Science*.

[40] Kessenich C. R. (2000). Osteoporosis and african-American women. *Women’s Health Issues*.

[41] Kannel W., Abbott R. D. (1987). Incidence and prognosis of myocardial infarction in women: The framingham study. *Coronary Heart Disease in Women*.

[42] Nazari M., Farmani S., Kaveh M. H., Ghaem H. (2016). The effectiveness of lifestyle educational program in health promoting behaviors and menopausal symptoms in 45-60-year-old women in marvdasht, Iran. *Global Journal of Health Science*.

[43] Malik E., Sheoran P., Siddiqui A. (2018). Health-promoting behaviors and menopausal symptoms: an interventional study in rural India. *Journal of Mid-life Health*.

[44] Mohamed H. A., Lamadah S. M. (2015). Improving women’s practices for reducing the severity of menopausal symptoms. *Journal of Nursing Education and Practice*.

